# ExoCAS-2: Rapid and Pure Isolation of Exosomes by Anionic Exchange Using Magnetic Beads

**DOI:** 10.3390/biomedicines9010028

**Published:** 2021-01-02

**Authors:** Hyunsung Kim, Sehyun Shin

**Affiliations:** 1 School of Mechanical engineering, Korea University, Seoul 02841, Korea; hyunsung24@korea.ac.kr; 2 Engineering Research Center for Biofluid Biopsy, Korea University, Seoul 02841, Korea

**Keywords:** exosome, isolation, cationic polymer, magnetic beads, ion exchange

## Abstract

Extracellular vesicles (EVs) are considered essential biomarkers in liquid biopsies. Despite intensive efforts aimed at employing EVs in a clinical setting, workable approaches are currently limited owing to the fact that EV-isolation technologies are still in a nascent stage. This study introduces a magnetic bead-based ion exchange platform for isolating EVs called ExoCAS-2 (exosome clustering and scattering). Owing to their negative charge, exosomes can easily adhere to magnetic beads coated with a polycationic polymer. Owing to the features of magnetic beads, exosomes can be easily processed via washing and elution steps and isolated with high purity and yield within 40 min. The present results confirmed the isolation of exosomes through analyses of size distribution, morphology, surface and internal protein markers, and exosomal RNA. Compared with the commercially available methods, the proposed method showed superior performance in terms of key aspects, including operation time, purity, and recovery rate. This highlights the potential of this magnetic bead-based ion exchange platform for isolating exosomes present in blood plasma.

## 1. Introduction

Nano-sized (30–160 nm) exosomes, discovered in 1983 [1,2], are a subset of extracellular vesicles (EVs). They are shed from almost all cell types in the human body into body fluids, such as blood, urine, saliva, milk, semen, etc. [3,4]. Exosomes envelop various functional biomolecules, including nucleic acids (DNA, mRNA, miRNA, non-coding RNA, lncRNA), lipids, proteins, carbohydrates, and metabolites [5,6,7]. Due to the protein–lipid structure of the EV membrane, the nucleic acids enclosed within these vesicles are well preserved from external damage in body fluids. Once considered to be cellular garbage packs, exosomes are now recognized as important mediators of intercellular communication, transporting diverse cargo to other cells. In fact, tumor cells are reported to release more exosomes than normal cells [8]. Exosomes are considered to be closely associated with the pathological mechanisms underlying various diseases, including cancer, neurodegenerative disorders, multiple sclerosis, and inflammatory diseases, by virtue of their role in intercellular communication [9,10,11,12,13,14].

Some features of tumor-derived exosomes make them attractive biomarkers for liquid biopsies, which could be used to help diagnose cancers earlier, monitor therapeutic responses, test for drug resistance, and guide selection of therapeutic strategies [15]. Despite the increasing potential utility of exosomes as liquid biopsies, EV research remains restricted by the current technical limitations of EV isolation [16,17,18]. In fact, EVs are challenging to analyze mainly because of their unique characteristics, that is, their nanoscale size, nearly neutral buoyancy, and excessive protein and lipid content in body fluids [16]. The current gold standard isolation method is ultra-centrifugation (UC), which is highly labor-intensive and time-consuming and has a poor yield. Many techniques have been developed for more efficient exosome separation, including size-, charge-, affinity-, and polymer precipitation-based techniques, as well as microfluidics techniques [19,20,21,22,23,24].

Unfortunately, these current technologies are not suitable for clinical applications because of their limitations with respect to purity and recovery rate. Thus, the development of innovative exosome isolation methods will lead to significant advances in the diagnosis and treatment of cancer. In the present study, we proposed an innovative mobile ionic exchange platform with magnetic beads called ExoCAS-2 (exosome clustering and scattering), which could potentially contribute significantly to studies on EVs, including research on EV-based molecular diagnostics, treatment monitoring, and drug delivery systems.

## 2. Materials and Methods

### 2.1. Working Priciples and Method of ExoCAS-2

**Figure 1:**
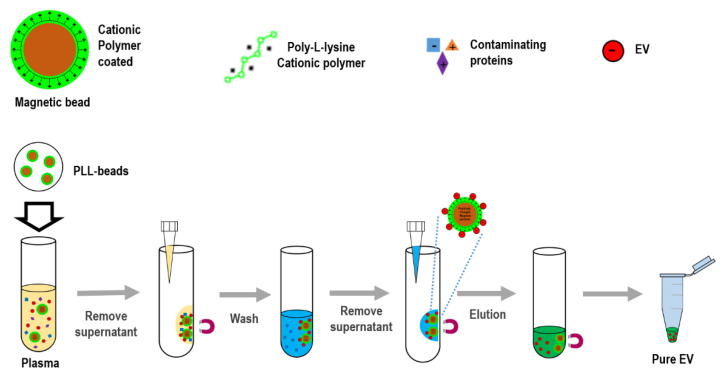
Schematic for extracellular vesicles (EV) isolation using cationic polymer-coated beads. Experimental procedure in which magnetic beads coated with cationic poly-L-lysine (PLL) polymer were mixed with anionic exosomes. Exosome-bound beads were washed and isolated via a magnetic feature.

To isolate exosomes from biofluids, the present study introduced an innovative platform (ExoCAS-2) containing charge-based ion exchange and magnetic bead-based manipulation. First, ExoCAS-2 uses polycationic polymer-functionalized magnetic beads (Figure 1). The poly-L-lysine (PLL, Sigma-Aldrich, St. Louis, MO, USA) polymer was coated on beads, which were inputted into the plasma. The plasma sample is best filtered in advance to ensure that particles larger than 0.8 μm are excluded from the supernatants (e.g., using Sartorius Minisart™ NML; cat. No. 16592). Owing to the polycationic characteristics of the PLL polymer, negatively charged EVs were combined with the positively charged PLL beads via electrostatic reactions. This binding process required a specific temperature (4 °C) and minimum incubation time (30 min) in a rocking platform mixer at 90 rpm. As the PLL-coated beads are in a mobile state in samples, the binding between beads and exosomes can be maximized by gentle mixing. In this process, several hundred nanoscale EVs were captured on the surface of the PLL-coated beads. After incubation for 5 min, a magnet was placed near the tube and the exosome-captured beads were collected within 2 min. The supernatant was then removed using a pipette. The supernatant should be removed carefully to avoid disturbing the exosome-bound beads.

Next, the exosome-bound beads were carefully washed to remove non-target proteins using a washing buffer (2 mL) with pH 6 to resuspend the exosome captured beads.

A magnet was used to attract the EVs-captured PLL-beads from the mixing solution; meanwhile, the unnecessary proteins were rinsed out. Bead-bound exosomes were then separated and collected using an elution buffer with salt at an appropriate concentration (NaCl 1 M, pH 7.0, 200 µL). In order to fully detach the exosome from the beads, the elution buffer was vortexed for 5 min at 1000 rpm. A magnet was then placed near the tube for 2 min to collect the magnetic beads. The supernatant, containing with pure EVs, was then collected with a pipette. The whole process was completed in 40 min, which consisted of incubating, washing, and eluting. Finally, large numbers of high-purity isolated EVs were sent for further molecular analysis and physical characterization analysis by Nano Sight (NTA) to measure the particle diameter.

### 2.2. Blood Sample Preparation

Plasma samples were purchased from Zen-Bio Inc. (Research Triangle, NC, USA). Large debris, which might be existed, was removed with mesh filtering (800 nm pore-size mesh). Unless otherwise specified, a plasma volume of 1 mL was used for downstream analysis.

### 2.3. Isolation of EVs

#### 2.3.1. Ultracentrifugation

Ultracentrifugation (UC) is a commonly used gold standard method for isolating EVs, even though it requires a time consuming process (t > 6 h) and labor intensive and repetitive manipulation. In this study, plasma was mixed with phosphate buffered saline (PBS) in a 1:1 ratio, and the mixture was centrifuged to remove residual cellular components (4 °C, 12,000× *g*, 30 min). The supernatant was transferred and repeated centrifugation under the same conditions. The supernatant was filtered with a syringe filter having 220 μm pore-size (Merck Millipore, Burlington, MA, USA). Then, the filtered supernatant was centrifugated with a high speed centrifuge (CP100WX; Hitachi, Tokyo, Japan) at 120,000× *g* and 4 °C for 2 h. After aspirating the supernatant, the pellet at the bottom was resuspended and washed in PBS at 120,000 *g* and 4 °C for 1 h and then finally resuspended in 50 μL of PBS.

#### 2.3.2. EV Isolation Using Commercial Products

ExoQuick™ exosome precipitation solution (EXOQ5A-1; System Biosciences, Palo Alto, CA, USA) was used for EV isolation. The experimental procedure is described in the manual. Briefly, the plasma sample was mixed with the ExoQuick solution, which is a PEG-based solution, followed by incubation of the mixture for 30 min at 4 °C. Post incubation, the mixture was centrifuged at 1500× *g* for 30 min, and the supernatant was carefully removed while leaving the pellet in the tube. After a second round of centrifugation at 1500× *g* for 5 min, all traces of the ExoQuick solution were removed, following which the pellet was resuspended in 200 μL of PBS.

Plasma exosomes were extracted using the exoEasyTM Maxi Kit (Qiagen, Valencia, CA, USA) according to the manufacturer’s instructions. Briefly, plasma filtration was performed to exclude particles with diameter greater than 0.8 µm. An equal volume of XBP buffer was added and mixed well, following which the suspension was transferred into an exoEasy spin column and centrifuged for 1 min at 500× *g*. The flow-through was removed. Next, 10 mL of XWP buffer was added and the suspension was centrifuged for 5 min at 5000× *g*. Following this, the flow-through was removed, the spin column was transferred to a new collection tube, 100 µL XE buffer was added, the mixture was centrifuged for 5 min at 5000× *g*, and the flow-through was collected as exosome resuspension B, which was named “exo-B.”

### 2.4. Analysis of Isolated Particles

#### 2.4.1. Zeta Potentials in EVs and Polycationic Polymer

The zeta potentials of EVs isolated using UC and ExoCAS-2 as well as that of microbeads conjugated with PLL were measured using a zeta potential analyzer (Zetasizer Pro; Malvern Panalytical, Malvern, UK). Because of the difficulty in resuspending the cluster with deionized water, the pellet-clustered PLL-beads (5 mg/mL) from 1 mL plasma were resuspended in 10 µL NaCl solution (1 M). Furthermore, 990 µL of deionized water was added to the resuspended solution.

#### 2.4.2. Cryo-Transmission Electron Microscopy Images

The EVs isolated using UC and the PLL bead method were transferred to a 20 nm mesh grid, which were then subjected to freezing incubation (−196 °C, 2 h) using VitrobotTM (FEI, Hillsboro, OR, USA). After the samples were prepared, the EVs were observed using transmission electron microscopy (Tecnai G2-F20, FEI, Hillsboro, OR, USA).

#### 2.4.3. Scanning Electron Microscopy (SEM) Images

After the plasma sample was ultracentrifuged, it was filtered using an anodic aluminum oxide membrane mounted in a gasket. Additionally, the PLL-coated bead sample was filtered using a membrane. The membranes were incubated in glutaraldehyde solution (Sigma-Aldrich, St. Louis, MO, USA) for 30 min. Following this, the membranes were sequentially rinsed with 25%, 50%, 75%, 90%, and 100% ethanol and incubated overnight at 37 °C in a dry oven. After the membranes were coated with Pt, EVs and cluster subjects on the membranes were observed using SEM (Quanta 250 FEG; FEI, Hillsboro, OR, USA).

#### 2.4.4. Nanoparticle Tracking Analysis (NTA)

For each NTA analysis, 1 mL of EV solution was isolated using UC, ExoQuick, exoEasy, or the PLL clustering method. Briefly, a PBS-diluted sample was placed in the assembled sample chamber of the NanoSight LM10 system (Malvern Panalytical, Worcestershire, UK), and the microparticles were focused using the fingerprint area as a reference. Video images of the EVs were recorded, and their mean sizes and concentrations were determined based on each dilution factor. Three independent replications were performed for each experiment.

### 2.5. Analysis of Proteins and Nucleic Acids

#### 2.5.1. Western Blot Analysis

Proteins from the isolated exosomes suspended in the elution buffer (200 μL) were denatured by heating at 95 °C in Laemmli buffer (Bio-Rad, Hercules, CA, USA) containing 2-mercaptoethanol (Sigma-Aldrich, St. Louis, MO, USA) for 10 min. Proteins were separated by electrophoresis on SDS-PAGE Mini-PROTEAN^®^ TGX™ Precast Gels (456–1035; Bio-Rad). Subsequently, the proteins were subjected to immunoblotting with rabbit polyclonal antibodies (1:2000 dilution), anti-TSG101 (ab125011), anti-CD81 (ab109201), anti-ALIX (ab186429), anti-CD9 (ab92726), and goat anti-rabbit IgG H&L (HRP) (ab205718) (Abcam, Cambridge, UK). The protein bands were visualized using an enhanced chemiluminescence reagent and the ChemiDoc™ XRS + System (Bio-Rad).

#### 2.5.2. Protein Contents

A PierceTM BCA Protein Assay Kit (#23225; Thermo Scientific, Waltham, MA, USA) was used for the assessment of purity. A standard curve (range 0–2000 μg/mL) was derived from nine serial dilutions of bovine serum albumin and a working reagent. Three replications were used for all samples and standard points. The samples (100 μL each) were mixed with 2.0 mL of the working reagent and incubated at 37 °C for 30 min. After cooling to room temperature, the difference between the absorbance values and the average absorbance of blank standard replicates at 562 nm, measured using a spectrometer (DS-11; Denovix, Wilmington, DE, USA) was determined, and the absorbance differences were converted to μg/mL based on the standard curve. If the protein concentration exceeded the upper limit of the standard curve (2000 μg/mL), the sample was diluted until the concentration reached a value within the standard range, and the final concentration was calibrated based on the dilution factor.

#### 2.5.3. RNA Analysis

RNA was isolated using the SeraMir Exosome RNA purification kit (RA806A-1, SBI, Palo Alto, CA, USA). All isolation processes were performed according to the manufacturer’s protocol. To quantify EV miRNA markers, the RNA eluate solution was subjected to reverse transcription using the TaqMan MicroRNA RT kit (4366596, Life Technologies, Carlsbad, CA, USA) and TaqMan Micro RNA Assays (4427975, Life Technologies, Carlsbad, CA, USA). TaqMan Universal Master Mix II, no UNG (4440040, Life Technologies, Carlsbad, CA, USA) was used, together with the miRNA hsa-let-7a-5p, ID 000377, and hsa-miR-142-3p, ID 000464. Further experiments were performed with RNA eluate using the Agilent Eukaryote Total RNA Pico chip on an Agilent 2100 bioanalyzer (Agilent Technologies, Santa Clara, CA, USA).

#### 2.5.4. miRNA Analysis with Microarray

The miRNA expression profile was analyzed using a microarray (Gen-Chip miRNA 4.0 array, Affymetrix Inc., Santa Clara, CA, USA). The data were compared using three different methods (ExoCAS-2, exoEasy, and UC). Total RNA (130 ng), which included tissue miRNA, was labeled with biotin using FlashTag. Samples labeled with the TM Biotin HSR RNA Labeling Kit (Affymetrix) were mixed into the Affymetrix miRNA micro-array using the GeneChip^®^ Hybridization Oven as directed by the manufacturer. The labeled RNA was heated to 99 °C for 5 min and subsequently to 45 °C for 5 min. RNA-array hybridization was performed using the Affymetrix^®^ 450 Fluidics station for 16 h with stirring at 48 °C at 60 rpm. The chips were cleaned and stained using the Genechip Fluids Station 450 (Affymetrix). Each chip was scanned using the Affymetrix GCS 3000 scanner (Affymetrix). The signal value was determined using the Affymetrix^®^ GeneChip^®^ Command Console software (Affymetrix Inc., Santa Clara, CA, USA).

## 3. Results

### 3.1. Cationic Polymer-Captured EVs

**Figure 2:**
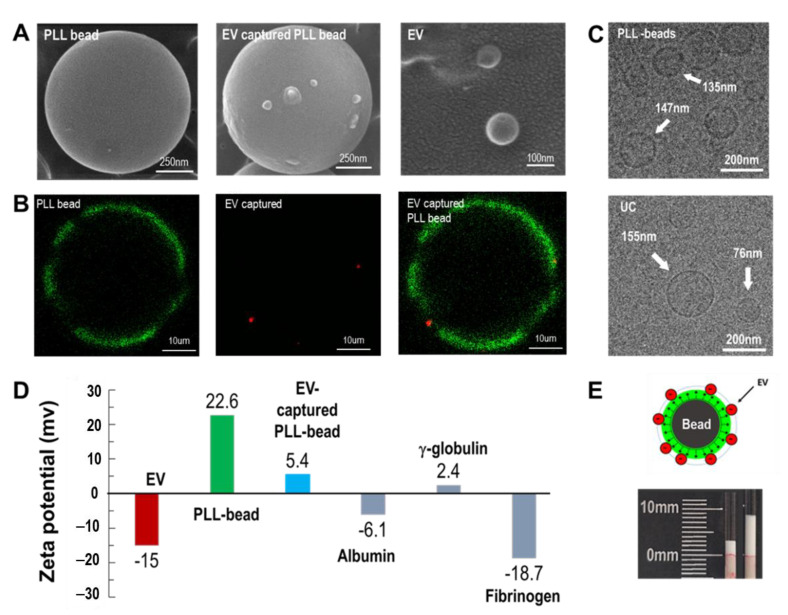
EV isolation using cationic polymer-coated beads. (**A**) SEM images of a PLL-coated bead, an EV-captured PLL bead, and isolated exosomes, respectively. The mean size of the magnetic beads was 950 nm. (**B**) Fluorescent images of PLL-coated magnetic beads (green color), exosome capture by PLL bead (red color), and the merged image. (**C**) Cryo-TEM images of EVs eluted from PLL beads and ultracentrifugation (UC). (**D**) Zeta potentials of exosomes, PLL beads, EV-bound PLL beads, and plasma proteins, including albumin, γ-globulin, and fibrinogen. (**E**) Volume-change analysis after incubating PLL beads with blood plasma.

Magnetic beads, exosome-captured beads, and isolated exosomes were visualized using SEM (Figure 2A). After examining various sizes of beads (0.5–40 μm), 1 μm beads were chosen and used for the entire study unless otherwise specified. Several exosomes were bound to the surfaces of PLL-coated magnetic beads. In addition, exosomes isolated from the magnetic beads were observed to be approximately 100 nm in size. Similar results were obtained by fluorescence microscopy (Figure 2B). The PLL-coated magnetic beads were visualized by staining with FITC, whereas exosomes were stained with anti-CD 63 (Alexa Fluor 647). For visualization of fluorescence, 40 μm beads were used. The green color indicates the PLL conjugated on the surface of a microbead, whereas the red color indicates the EVs captured on the PLL polymer. EVs isolated from ExoCAS-2 and UC were visualized using TEM. Both methods yielded vesicles of nearly the same size, ranging from 70–180 nm in diameter (Figure 2C).

The protocol of the present exosome isolation involves a typical anionic exchange via PLL-coated magnetic bead surfaces. The zeta potentials of EVs were highly negative (−15.0 mV) owing to the anionic phospholipid bilayer of EVs (Figure 2D). The PLL-coated beads yielded a highly positive zeta potential (22.6 mV) owing to the cationic hydrophilic amino group of PLL. Following exosome capture of the bead surface, the zeta potential of the exosome-bound beads became weakly positive (5.4 mV). It is worth noting that the zeta potential of PLL is 42.4 mV, as reported in a previous study [24]. The strong cationic characteristic of PLL slightly decreased after it bound to the bead and further decreased as the EVs bound to it. Meanwhile, many plasma proteins have a negative zeta potential. The zeta potentials of albumin, γ-globulin, and fibrinogen were identified as −6.1, 2.4, and −18.7, respectively. Owing to the operating principle of anion exchange in the current study, unwanted anionic plasma proteins may co-exist with target exosomes and thus should be carefully removed during washing.

It is worth noting that there was an apparent volume change of beads before and after incubation of the PLL-beads in plasma (Figure 2E). Attachment of exosomes to the beads resulted in an increase in the effective bead size. Depending on the incubation time and ambient temperature, the bead volume increased significantly. Similar to the results obtained in a previous study, incubation at 4 °C accelerated the binding of exosomes to the PLL polymer [24]. Additionally, at 30 min of incubation, the volume increase was fully saturated. The detailed results are shown in Figure S1.

### 3.2. Comparison of Recovery Yield and Protein Concentration

**Figure 3:**
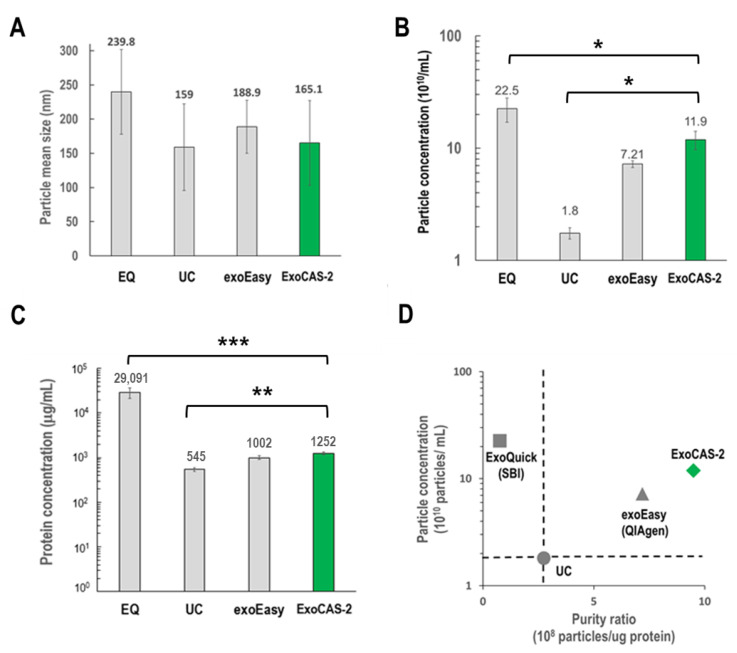
Comparison of EV isolation methods (EQ, UC, exoEasy, PLL-beads) using NTA, and BCA. (**A**) Mean size. (**B**,**C**) Particle concentration and protein concentration (*: *p* < 0.05, **: *p* < 0.01, ***: *p* < 0.001). (**D**) Normalized purity ratio (particle/protein ratio), using UC as a reference standard for both particle and purity ratio (dotted line). EQ: ExoQuick (SBI), UC: ultracentrifugation, exoEasy (Qiagen), ExoCAS-2: present research.

Exosomes isolated using ExoCAS-2 were carefully examined for size distribution, morphology, surface and internal protein markers, and exosomal RNA, and the results were compared with those of available methods including ultracentrifugation (UC), EQ (exoQuick, SBI), and exoEasy (Qiagen). Nanoparticle tracking analysis (NTA) was used to compare the average sizes of the exosomes obtained using the four different methods (Figure 3). No significant differences in exosome size were observed among the methods except for ExoQuick. However, there were significant differences in particle concentration and protein contamination among the exosomes isolated using the different methods (Figure 3B,C).

ExoQuick showed the maximum particle concentration (22.5 × 10^10^/mL), which was 12.5 times higher than that of UC (1.8 × 10^10^/mL) and nearly two-fold that of the present ExoCAS-2. However, ExoQuick use was associated with severe protein contamination, which was more than 50-fold higher than that of UC; protein contamination with the present method was approximately 2.3-fold that of UC. With respect to the purity ratio, defined as the ratio of particle concentration to protein concentration, the ExoCAS-2 method showed the highest value among the four different methods.

### 3.3. Comparison of Proteins and microRNA Extracted from EVs

**Figure 4:**
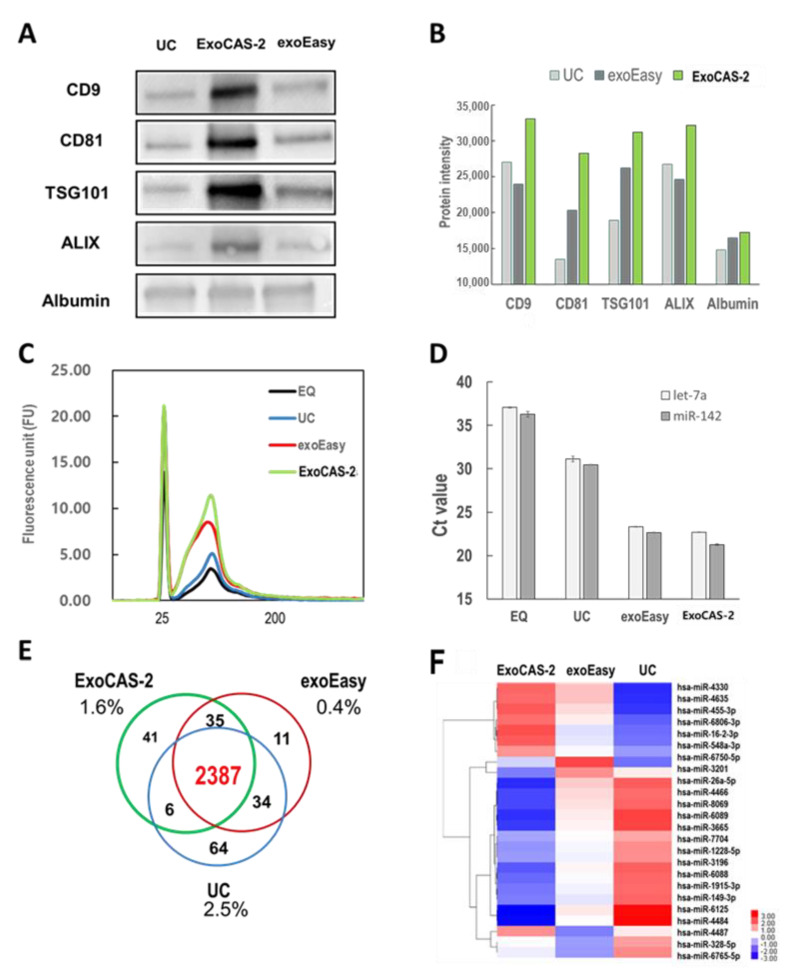
Comparison of EV isolation methods (EQ, UC, exoEasy, PLL beads) using bioanalyzer, western blot, RT-qPCR, and microarray. (**A**) Western blot for investigating protein markers of EVs. (**B**) Western blot analysis for proteins extracted from EVs. (**C**) Analysis of total exosomal RNA using bioanalyzer with Agilent eukaryote total RNA pico chips. (**D**) Exosomal miRNAs (hsa-let-7a-5p, hsa-miR-142-3p) measured by RT-qPCR. (**E**,**F**) Venn diagram and heatmap depicting gene expression by microarray analysis. EQ: ExoQuick (SBI), UC: ultracentrifugation, exoEasy (Qiagen), ExoCAS-2: present research.

In order to confirm whether the isolated EVs were exosomes, the presence of protein markers was investigated in isolated vesicles using western blotting (Figure 4A). Tetraspanins CD9 and CD81 frequently serve as the surface markers of exosomes, whereas Alix (ALG-2-interacting protein X), and TSG101 (tumor susceptibility gene 101 protein) do as the inner protein markers of exosomes [25,26,27]. These four proteins were examined as reference markers for exosome identification in the present study. In addition, this study further examined albumin as a contaminating marker of plasma proteins. Among the three methods, the ExoCAS-2 method showed intense bands for the reference protein markers (ALIX, TSG101, CD9, and CD81). Band intensities were further analyzed quantitatively. Depending on the intensity levels of the exosome protein markers, the methods can be sorted in the following order: ExoCAS-2 > UC > exoEasy. Based on the presence of surface (CD9 and CD81) and inner protein (TSG101 and ALIX) markers, the isolated vesicles were confirmed as exosomes. The band intensity of albumin was low, but was detected in all methods, even though there was no apparent difference among the different methods. The band intensities of protein markers were replotted quantitatively, as shown in Figure 4B.

The RNA in the exosomes was also investigated in the present study (Figure 4C). EVs were isolated from the same plasma with four different methods, and RNA was extracted using the SeraMir Exosome RNA Purification Kit (SBI, Palo Alto, CA, USA). After RNA extraction, total RNA was measured using an Agilent Bioanalyzer 2100 (Agilent Technologies, Santa Clara, CA, USA). ExoCAS-2 showed the highest value of total RNA, and EQ showed the lowest value. Among the RNAs extracted from exosomes, some were further analyzed by RT-qPCR (Figure 4D). The Ct values for both miRNAs yielded similar values but were significantly different depending on the isolation method. These values for UC were 31.1 and 30.5 for hsa-let-7a-5p and hsa-miR-142-3p, respectively. Those for EQ were fairly high, which may be due to severe protein contamination. As expected from the purity ratio, the ExoCAS-2 method yielded the lowest Ct values (22.7 and 21.2), whereas exoEasy yielded the second-lowest values (23.3 and 22.6) among the four methods.

Using the Gen-Chip miRNA 4.0 array (Affymetrix Inc., Santa Clara, CA, USA), a total of 2578 miRNAs were identified in the extracted EVs using three different methods and analyzed with a Venn diagram (Figure 4E). Surprisingly, 92.6% of the overall exosomal miRNAs were found regardless of the isolation method used [20,28]. In the case of 26 miRNA genes that showed differences in expression, no specific miRNA related to cancer existed (Figure 4F). The percentage of miRNAs uniquely identified by each method never exceeded 2.5%. Information regarding the effects of PLL incubation conditions (Figure S1) and particle size (Figure S2) is included in the supplementary information.

### 3.4. Optimization of Washing and Elution Processes

**Figure 5:**
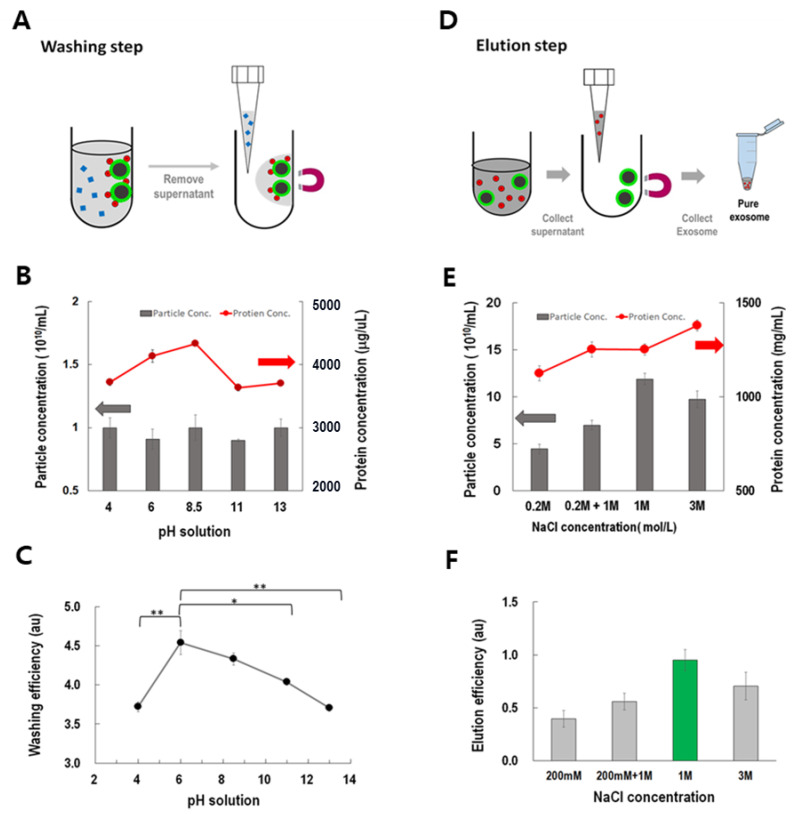
Optimization of washing and eluting processes under the current PLL-coated magnetic bead-based ion exchange method. (**A**) Washing step to remove undesired proteins. (**B**,**C**) Particle concentration, protein concentration, and washing efficiency (protein/particle ratio) in the washing step (*: *p* < 0.05, **: *p* < 0.01). (**D**) Elution step to collect pure exosomes. (**E**,**F**) Particle concentration, protein concentration, and elution efficiency (particle/protein ratio) in the elution step. (Grey arrow: the coordinates of the bar in the figure; Red arrow: the coordinates of the line in the figure).

ExoCAS-2 is based on the principle of anion exchange. First, the unwanted proteins were removed from the beads, as shown in Figure 5A. Negatively charged proteins should be removed without affecting the EVs. When the buffer pH is equal to the isoelectric point (pI), the protein charge becomes neutral and can be easily eluted from the ion exchange resin. Adjusting the pH of an elution buffer below pI induces positive charge of protein and vice versa. However, various plasma proteins have a wide range of isoelectric points (pI) from to 5–9 [29,30].

Buffers with varying pH were examined for their potential as protein washes (Figure 5B). The pH was adjusted with a fixed concentration of acetic acid (29 nM) and various concentrations of sodium hydroxide (6.135 nM). Interestingly, the washed proteins showed a peak near pH 8.5 in the washing buffer, whereas those derived from the washed particles did not show any apparent difference at the examined pH values. Considering the washing efficiency, defined as the ratio of the concentration of washed proteins to the total number of particles washed, the buffer at pH 6 showed the highest washing efficiency and had a pH value significantly different from the other pH values (Figure 5C).

After the washing step, all of the EVs were detached from the PLL magnetic beads through the ionic exchange scheme (Figure 5D). After examining various salts (Na_2_SO_4_, (NH_4_)_2_SO_4_, KCl, CH_3_COONH_4_, and NaCl), sodium chloride was carefully selected because of the anionic exchange capacity of chloride. Solutions with various concentrations of NaCl (200 mM–3 M) and gradient methods (200 mM + 1 M) were examined [31]. With increasing NaCl concentration, the recovered particles show a maximum at 1 M rather than 3 M (Figure 5E), which can be explained by the salting-out effect [25]. However, the concentrations of proteins in the eluted buffer increased with NaCl concentration. Thus, considering eluting efficiency, defined as the ratio of the number of particles to the concentration of proteins in the eluted buffer, 1 M of NaCl buffer showed the highest value among all buffers. When the NaCl concentration was 1 M, most exosomes were extracted (Figure 5F).

## 4. Discussion

Despite the clinical significance of EVs, including early detection and therapeutic monitoring of cancer, EV isolation and purification processes have been a bottleneck for clinical applications due to technical difficulties. Furthermore, sample preparation is crucial to the success of downstream analyses of surface protein markers as well as internal nucleic acids, including micro RNA. If not performed properly, it can aggravate the entire analysis project and be quite costly in terms of money and time. In response, innovative methods should be implemented with various requirements such as delivering higher quality prepared samples, requiring less hands-on time, and increasing speed, throughput, reproducibility, reliability, and laboratory efficiency. To meet these unmet technical needs, ExoCAS-2 was developed, which can satisfy the above requirements.

First, ExoCAS-2 higher-quality prepared samples in terms of purity and quantity. In fact, ExoCAS-2 provides 6.6- and 1.7-fold higher yields compared with those of UC and exoEasy, respectively. Second, ExoCAS-2 provides excellent reproducibility, owing to the well-controlled bead size, uniform polymer coating, and magnetic manipulation. Unique batch-to-batch repeatability (typical CV < 5%) leads to high quality of results. Fourth, the polycationic polymer coated on the surface of microparticles effectively captures EVs through charge interaction and freely-moving microparticle kinetics. These features enable increases to the isolation performance including yields and purity compared with other methods. The entire process of EV isolation can be completed within 40 min, including the incubation step. In addition, magnetic handling provides easy handling with either manual or automated methods. Moreover, based on the unique characteristics of ExoCAS-2, it is potentially scalable for handling sample volumes from 100 μL to 50 L.

However, the proposed method has certain limitations. For instance, urine is commonly used to diagnose prostate cancer. However, the current method cannot be applied owing to the high concentration of chloride ions in urine. Chloride ions are the salt ions primarily exchanged with the captured exosomes. Therefore, the chloride ions in urine tend to strongly bind to the PLL beads and capture the space occupied by exosomes. This is possibly the most significant drawback of charge interaction methods for isolation of EVs from biofluids.

## 5. Conclusions

ExoCAS-2 presented in this study efficientlly isolated and examined characteristic of EVs from blood plasma. The main features of ExoCAS-2 come from magnetic, mobile, and microparticle-based ion exchange resins. The mobile resin can freely move and recruit counterionic objects in the liquid phase. Since conventional ionic exchange adopts a once-through flow system with stationary resins, target ions flow away with a decreasing flow rate. Owing to microfabrication technology and its characteristics, uniform size and significantly increased surface area are key to capturing the target objects. Additionally, magnetic separation is surprisingly easy. Furthermore, ExoCAS-2 can be integrated with an automated microfluidic system (PIBEX™), which has been developed for the extraction of cell-free DNA from plasma [26,27]. Exosomal nucleic acids can be directly obtained using a kit with automated microfluidic operation. As a follow-up study, a wider range of sample fluids will be tested, including urine, saliva, sputum, and exhale breath condensates.

The current method is expected to be applied to basic research and clinical trials that require isolation of EVs. As ExoCAS-2 exhibits high performance for EV isolation, it could play a significant role in the advancement of EV-based basic research, including research on biomarker discovery and drug delivery system development, as well as clinical applications such as molecular diagnostic methods and treatment monitoring strategies.

## Data Availability

Not applicable.

## Supplementary Materials

The following are available online at https://www.mdpi.com/2227-9059/9/1/28/s1, Figure S1: Changes in microbead volume before and after incubating beads with plasma. Figure S2: Comparison of EV isolation performance for two different bead sizes.
