# Supplementary material for: ExoCAS-2: Rapid and Pure Isolation of Exosomes by Anionic Exchange Using Magnetic Beads
Source: Biomedicines. 2021 Jan 2;9(1):28. doi: 10.3390/biomedicines9010028 (PMC7824726; doi:10.3390/biomedicines9010028)
Supplement: Supplementary file 1 [file biomedicines-09-00028-s001.pdf]

biomedicines

Article

ExoCAS-2: Rapid and Pure Isolation of Exosomes by Anionic Exchange using Magnetic Beads

HyunSung Kim[1], and Sehyun Shin[1, 2,*]

[1] School of Mechanical engineering, Korea University, Seoul, Korea; hyunsung24@korea.ac.kr (H. K.)
[2] Engineering Research Center for Biofluid Biopsy, Seoul, Korea; lexerdshin@korea.ac.kr (S.S.)
* Correspondence: lexerdshin@korea.ac.kr; Tel.: +82-10-4506-2825

* To whom correspondence should be addressed:

Sehyun Shin

Telephone: (82) 2-3290-3377

E-mail: lexerdshin@korea.ac.kr

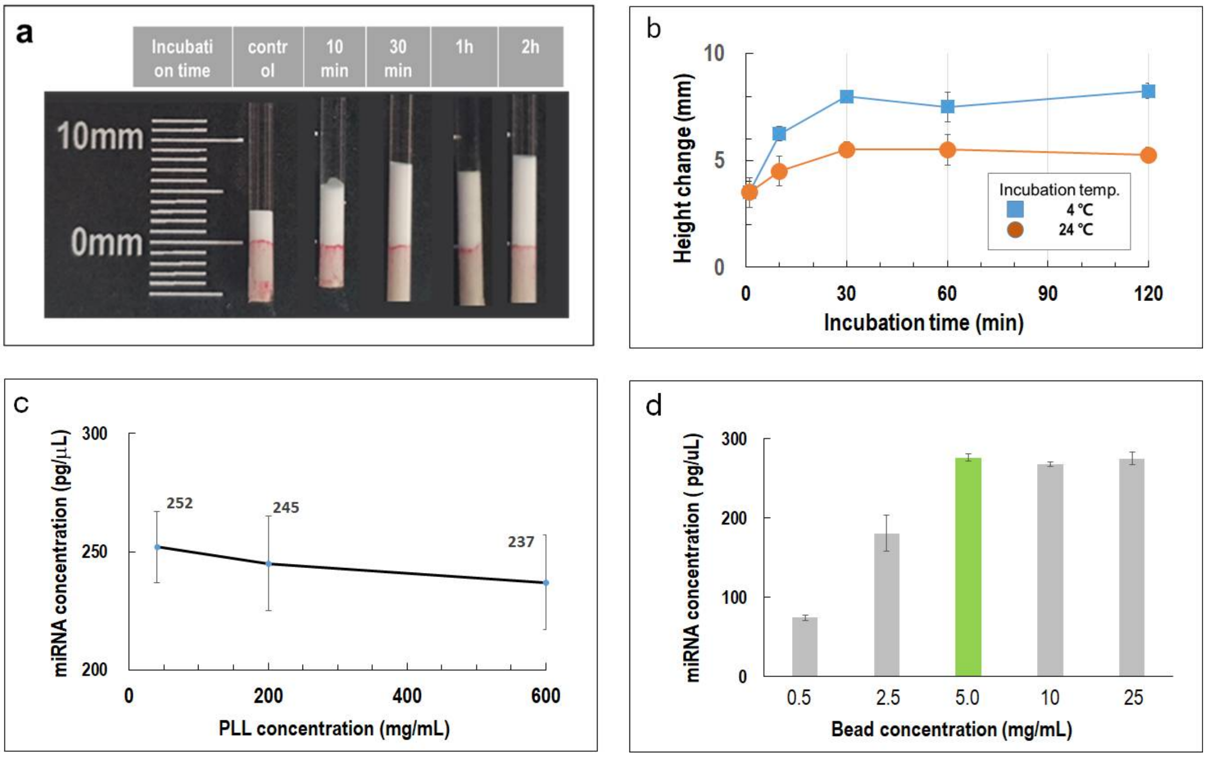

Figure S1 Changes in microbead volume before and after incubating beads with plasma. a) Volume changes of microbeads incubated in the same aliquot of plasma with varying incubation times; b) Effect of PLL concentration on height changes of EV capture for two different incubation times; c) Effect of PLL concentration on extracted miRNA from EVs; d) Effect of bead concentration on extracted miRNA from EVs

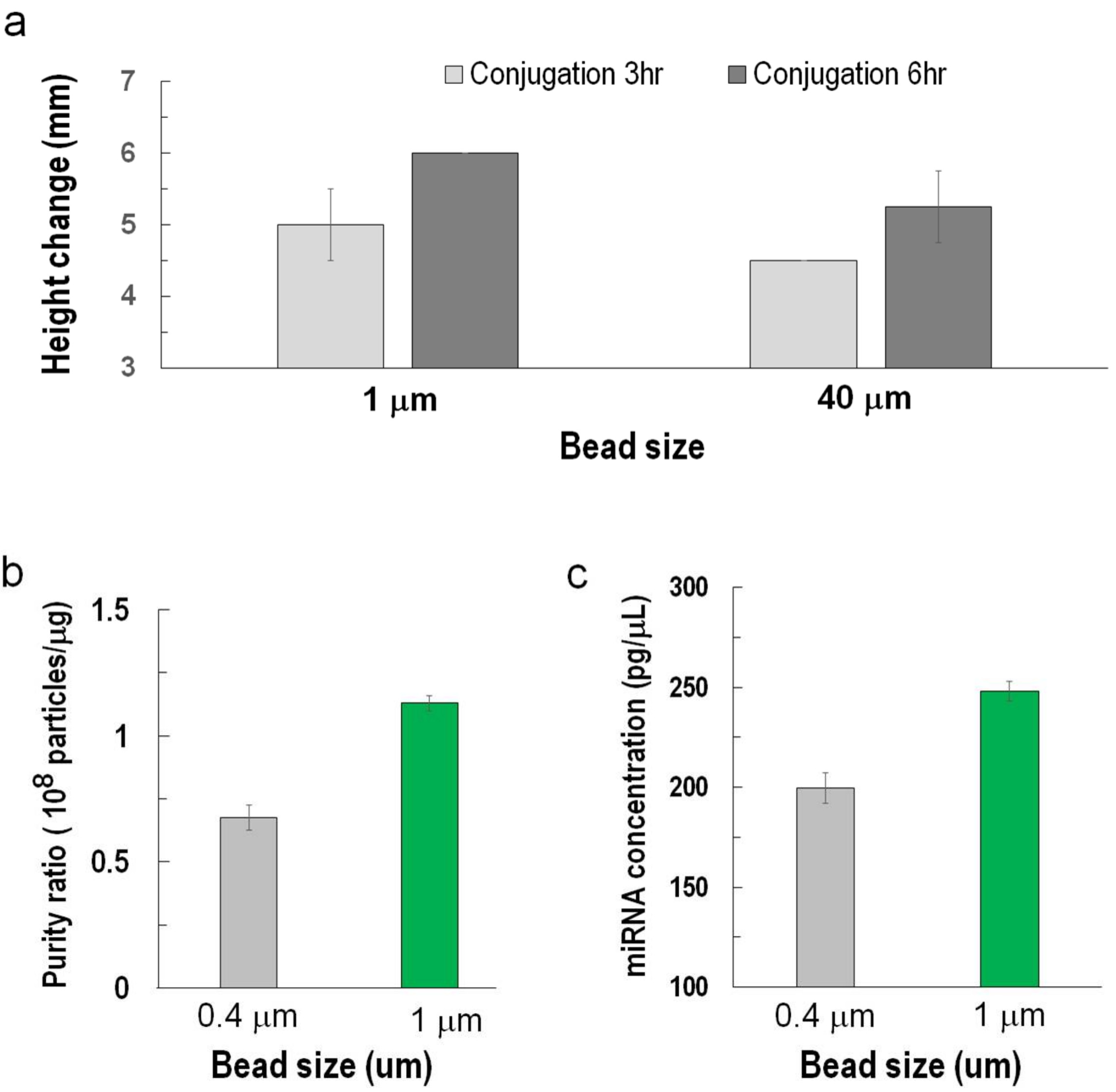

Figure S2 Comparison of EV isolation performance with two different bead sizes a) Changes of microbead volume before and after incubating beads in a plasma. Microbeads incubated in the same, aliquot plasma with varying PLL-conjugation time and bead size. b) - c) Effect of bead size on EV capture purity ratio and miRNA concentration
